# Genomic Sequencing Results Disclosure in Diverse and Medically Underserved Populations: Themes, Challenges, and Strategies from the CSER Consortium

**DOI:** 10.3390/jpm11030202

**Published:** 2021-03-13

**Authors:** Sabrina A. Suckiel, Julianne M. O’Daniel, Katherine E. Donohue, Katie M. Gallagher, Marian J. Gilmore, Laura G. Hendon, Galen Joseph, Billie R. Lianoglou, Jennifer M. Mathews, Mary E. Norton, Jacqueline A. Odgis, Alexis F. Poss, Shannon Rego, Sarah Scollon, Tiffany Yip, Laura M. Amendola

**Affiliations:** 1 The Institute for Genomic Health, Icahn School of Medicine at Mount Sinai, New York, NY 10029, USA; katherine.donohue@mssm.edu (K.E.D.); jacqueline.odgis@mssm.edu (J.A.O.); 2 Department of Genetics, University of North Carolina at Chapel Hill, Chapel Hill, NC 27599, USA; julianne_odaniel@med.unc.edu; 3 Department of Pediatrics, Children’s Hospital at Montefiore, New York, NY 10467, USA; katie.gallagher@invitae.com; 4 Center for Health Research, Department of Translational and Applied Genomics, Kaiser Permanente Northwest, Portland, OR 97227, USA; Marian.J.Gilmore@kpchr.org; 5 Department of Pediatrics, University of Mississippi Medical Center, Jackson, MS 39216, USA; lhendon@umc.edu; 6 Department of Humanities and Social Sciences, University of California, San Francisco, CA 94143, USA; galen.joseph@ucsf.edu; 7 Institute for Human Genetics, University of California, San Francisco, CA 94143, USA; Billie.Lianoglou@ucsf.edu (B.R.L.); mary.norton@ucsf.edu (M.E.N.); shannon.rego@ucsf.edu (S.R.); tiffany.yip@ucsf.edu (T.Y.); 8 Department of Pediatrics, University of North Carolina at Chapel Hill, Chapel Hill, NC 27599, USA; muelljm@email.unc.edu (J.M.M.); alexis.poss@unc.edu (A.F.P.); 9 Department of Pediatrics, Baylor College of Medicine, Houston, TX 77030, USA; sxscollo@texaschildrens.org; 10 Division of Medical Genetics, University of Washington, Seattle, WA 98195, USA; LAmendola@medicine.washington.edu

**Keywords:** genomic sequencing, exome sequencing, genome sequencing, genetic counseling, return of results, underrepresented populations

## Abstract

Genomic sequencing results need to be effectively communicated across all populations and practice settings. Projects in the Clinical Sequencing Evidence-Generating Research (CSER) consortium enroll diverse racial/ethnic and medically underserved participants across various clinical contexts. This article explores a set of CSER results disclosure cases to expand the evidence base on experiences returning genomic results. Case details were collected using a structured set of questions. We identified common themes in the case set, and assessed challenges and strategies in achieving six relevant results disclosure objectives. CSER-affiliated patient/community stakeholder impressions of the findings were solicited via video conference calls. Seventeen cases across six CSER projects were included. Case themes sorted into four categories: (1) factors influencing participant understanding, (2) participant emotional response, (3) disease burden, and (4) logistical challenges. Challenges meeting results disclosure objectives included a lack of dialogue, health literacy level, unexpected findings, and complex concepts. Strategies were consistent with traditional genetic counseling practice, but also highlighted approaches being evaluated in CSER projects. Patient/community stakeholders supported the identified themes and provided additional suggestions to improve patient understanding and engagement. These experiences add valuable insights into adapting genomic results disclosure practices to best serve all patient populations.

## 1. Introduction

Genomic sequencing tests are increasingly utilized in clinical medicine and research settings. To fully realize the benefits of genomic sequencing, results need to be effectively communicated to patients and research participants. Processes and experiences returning results in the early and ongoing implementation of genomic sequencing have been explored through federally funded consortia [1,2,3], as well as sizable biobank-based community health initiatives [4]. During the early implementation of genomic sequencing tests into clinical practice, research from the Clinical Sequencing Exploratory Research (CSER)-phase one consortium identified themes in the disclosure of genomic results, such as challenges returning large amounts of information, adherence to follow-up recommendations for secondary findings, and the appropriate interpretation of negative results [5]. Recommendations from these experiences included implementing a multi-visit return model, the need for educational resources for patients and non-genetic providers, and the importance of managing patient expectations [3,6].

There is, however, a paucity of evidence related to experiences and outcomes in genomic sequencing research in ancestrally diverse and medically underserved groups, particularly non-European ancestral populations [7,8]. Large research initiatives that actively address this disparity are underway, including the work of the All of Us Research Program [9] and the Clinical Sequencing Evidence-Generating Research (CSER) consortium [10], albeit with different aims. Funded in 2017, this second phase of the CSER consortium is a network of seven clinical research projects investigating the integration of genomic sequencing into the clinical care of diverse patients across a range of disease states and healthcare settings. CSER consortium projects aim to enroll participants from diverse racial/ethnic populations, underserved populations, or populations who experience poorer health outcomes [11]. These projects enroll participants in prenatal, pediatric, and adult settings with a range of phenotypes, including neurologic, immunologic, and cardiac conditions, congenital anomalies, dysmorphic features, and hereditary cancer risk [10]. While CSER consortium projects differ in target age group, clinical setting, and disease state, their commonalities include performing indication-based genomic sequencing tests and returning diagnostic and secondary findings to participants.

The current phase of the CSER consortium provides the opportunity to build and expand the evidence base surrounding experiences returning genomic results. Providers have now had several years of experience with genomic sequencing tests. Additionally, results from CSER projects are disclosed in the context of genomic implementation research studies enriched for diverse racial/ethnic and medically underserved participants and are exploring different results disclosure approaches [10]. Finally, CSER offers the opportunity for engagement between researchers and patient/community stakeholders, which has been shown to provide valuable community perspective in other genomic research settings [12].

This project was conducted by a subgroup of members of the CSER consortium Education and Return of Results (Edu/RoR) Working Group. We examined a set of representative result return cases from CSER consortium projects and obtained patient/community stakeholders’ input. The findings presented here inform best practices for genomic results disclosure by sharing experiences working with diverse populations across various clinical contexts.

## 2. Materials and Methods

We collected a set of results disclosure cases that represent experiences returning genomic results to participants enrolled in CSER consortium projects. Providers involved in results disclosure and who are members of the CSER Edu/RoR Working Group contributed cases. Case collection continued until all result categories (positive, negative, and uncertain primary results and positive secondary findings) were represented. All submitted cases were included in the case set. Case details were collected using a standardized case summary form adapted from a previous study [5]. The case summary form asked providers to describe the indication for testing, test performed, result(s) type, reflections associated with the cases’ psychological and social components, and specific aspects of the case of interest related to returning results to CSER participant populations. We also collected the sex and self-reported ancestry of the proband, for adult participants, or parent(s) of the proband, for pediatric participants. For this paper, participants are defined as the proband or parents of the proband enrolled in the CSER project. All participant experiences described were based on the provider’s perceptions of participant understanding, emotions, and reactions. Participants described in these cases are enrolled in Institutional Review Board-approved research protocols at each corresponding CSER project where informed consent was obtained.

Two co-authors (L.M.A., S.A.S.) read all submitted cases and identified an initial set of themes. Themes were refined through an iterative process of discussion and revisions carried out via calls and emails with the CSER Edu/ROR subgroup. The final set of themes for each submitted case was agreed upon by the provider who submitted it. Related themes were then categorized by two co-authors (L.M.A., S.A.S.).

We next utilized the Practice-Based Competencies (PBCs) for Genetic Counselors to identify objectives related to results disclosure in these clinical genomics research settings [13]. The six results disclosure objectives were: (1) establish a mutually agreed upon genetic counseling agenda; (2) identify, assess, and empathically respond to patient-participant concerns; (3) facilitate informed decision-making and adaptation to genetic risks; (4) effectively educate clients about a wide range of genetics and genomics information; (5) apply genetic counseling skills in a culturally responsive and respectful manner; and (6) adapt genetic counseling skills for varied service delivery models. Providers who submitted a case identified the challenges they encountered and/or strategies they used during results disclosure to meet the defined objectives. Cited strategies and challenges were independent events; a strategy used to meet an objective did not need to address a cited challenge directly. Two co-authors (L.M.A., S.A.S.) sorted and grouped the challenges and strategies identified across the case set with their corresponding objectives, which were then reviewed and finalized by the subgroup.

**Figure 1:**
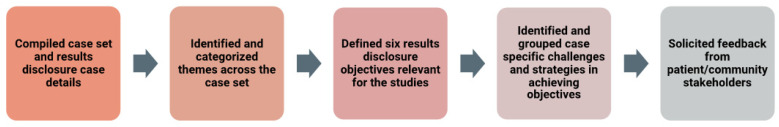
Project workflow. This project occurred in five stages: (1) compiling a case set, (2) identifying themes across the case set, (3) defining results disclosure objectives that were related to the Clinical Sequencing Evidence-Generating Research (CSER) projects, (4) identifying challenges and strategies in achieving the results disclosure objectives, and 5) soliciting feedback from CSER patient/community stakeholders.

Lastly, we conducted a series of consultations with a group of patient/community stakeholders active in the CSER consortium Patient, Community, and Clinical Stakeholder Engagement Working Group. The purpose of these consultations was to solicit feedback on the themes, challenges, and strategies in returning results identified by this project. Over the course of two video conference calls with a total of six patient/community stakeholders, we presented the background and goals of the project, the results disclosure objectives, and two example cases with their corresponding themes, challenges, and strategies. The two example cases were chosen because they included many of the themes and challenges identified in the case set. Patient/community stakeholder feedback and recommendations were compiled and summarized by the three co-authors who facilitated discussion on the conference calls (L.M.A., S.A.S., J.M.O.). See Figure 1 for the project workflow.

## 3. Results

### 3.1. Case Set and Themes

Seventeen cases across six CSER projects were compiled from twelve providers involved in disclosing results. One CSER project was not able to contribute cases, due to provider time constraints, and not all providers in the CSER Edu/ROR Working Group submitted a case. Distinct and shared themes were identified across the 17 cases (Table 1).

The case set represents all three categories of primary clinical results, including positive (4), uncertain (9), and negative (2) cases, a positive secondary findings case, and a case that reported a chromosome anomaly that was unrelated to the indication for testing. Cases included two adult probands; both were Hispanic/Latinx females. The remaining 15 cases included 22 parents of pediatric probands (seven mother–father dyads and eight mothers). Self-reported parental race/ethnicity was White (11), Hispanic/Latinx (7), Black (3), and multiple race/ethnicities (1, White and American Indian).

For the majority of cases (14), results were disclosed by a genetic counselor only. A genetic counselor and medical geneticist pair disclosed two results, and a neonatologist returned one result. Eleven results were returned in-person, three were returned by telephone, and three were returned first by telephone followed by an in-person appointment due to study design (1), accommodations for a prenatal participant’s delivery date (1), and to provide additional follow-up (1). Apart from a single case, results were returned by providers practicing in academic medical centers.

**Figure 2:**
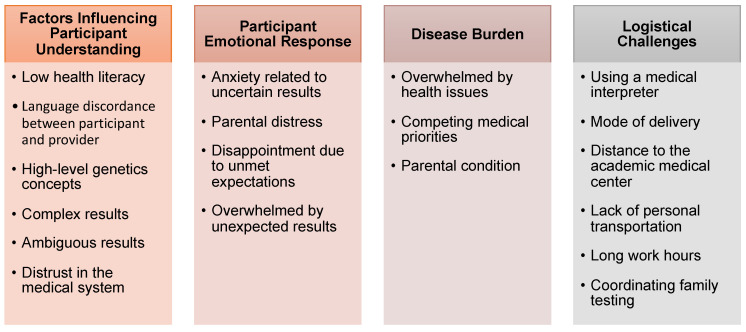
Categorized themes are highlighted in the case set.

Themes from the case set sorted into four main categories (Figure 2).

(1) Factors influencing participant understanding: Low health literacy and a language discordance between participant and provider were common themes in this category. The complexity of genetic information, generally, and the participants’ results, specifically, added an additional barrier to understanding. Providers described some cases involving ambiguous or discordant results as complicated and especially difficult for participants to understand. In one case, the participant’s distrust in the medical system affected how the results were believed and understood.
(2) Participant emotional response: Among cases with uncertain results, participants experienced anxiety and distress related to the results. Participant’s emotional responses affected how well they understood and adjusted to their result. Some participants who received unexpected results had trouble coping with the information. Additionally, a subset of participants had preset expectations for the testing, which led to participant disappointment for at least one of the cases with negative results.
(3) Disease burden: Some participants had multiple competing medical priorities and/or were overwhelmed by health problems and their associated medical management. Additionally, some parents of children enrolled in these studies had their own health condition(s), including parents with intellectual disability and a parent with vision impairment, which influenced how results were delivered.
(4) Logistical challenges: Logistical challenges experienced by participants complicated the return of results. For example, long work hours, lack of transportation, and distance from the medical center delayed return of results, which was particularly problematic in the context of a prenatal setting when disclosure timing can be important for pregnancy management. One participant could not travel to the institution for an in-person appointment because of a lack of social support and transportation. Additionally, providers noted that using a medical interpreter and delivering results by telephone complicated their assessment of patient coping.

### 3.2. Challenges and Strategies in Meeting Results Disclosure Objectives

Table 2 displays identified challenges and strategies grouped under the related results disclosure objective. The number of cases for which providers cited a challenge and/or strategy for each objective is included in the table.

Objective 1—Establish a mutually agreed upon genetic counseling agenda: Providers noted that participants harbored expectations for the testing that were not met, and some participants were left with unanswered questions, which impacted the sessions. For example, one participant sought answers about their child’s long-term health outcome; however, the ambiguous result could not provide such information. Another participant seemed disappointed that their child’s uninformative results did not support the need for additional therapies. Providers identified strategies they used to meet this objective, such as eliciting expectations and concerns upfront, and asking direct questions to establish the agenda.

Objective 2—Identify, assess, and empathically respond to participant concerns: Challenges associated with meeting this objective included health literacy level, emotional state, level of trust in the medical system, and minimal dialogue between the participant and the provider that led to difficulties assessing emotional needs. Additionally, for one case, the participant was not ready to receive the results, due to competing health concerns. In two cases, participants’ emotional needs were not met. Strategies, including active listening, evaluation of individual experiences and beliefs, and normalization, were utilized to meet this objective. Providers also addressed participants’ concerns through acknowledgment and affirmation.

Objective 3—Facilitate informed decision-making and adaptation to genetic risks: Participants had a limited understanding of complex genomic concepts, which presented a challenge in helping participants adapt to their results. For a subset of cases involving variants of uncertain significance, the uncertainty of the results and lack of concrete information were cited as challenges. Additionally, unexpected findings and uncertainty about how a given variant was inherited influenced participants’ decision-making and adaptation to their results. Providers did not indicate any particular strategies used during the results disclosure session to address this objective.

Objective 4—Effectively educate clients about a wide range of genetics and genomics information: Providers cited the highest number of cases with challenges related to this objective. A language discordance between the participant and provider and participant low health literacy were mentioned as challenges in providing effective education. The lack of concrete information with uncertain results was also identified as challenging. Providers cited specific strategies to achieve this objective, including providing information verbally and in writing, using images to explain concepts, repeating information when necessary, providing personalized result booklets, using educational tools, simplifying the information, and incorporating teach-back.

Objective 5—Apply genetic counseling skills in a culturally responsive and respectful manner: Providers did not report any challenges with meeting this objective; however, specific strategies were cited in two cases. These strategies included prioritizing participants’ needs and wants, utilizing both in-person and telephone interpreters, and including research personnel who have rapport with the participant as interpreters during the session.

Objective 6—Adapt genetic counseling skills for varied service delivery models: Challenges in meeting this objective included conveying complex concepts over the telephone, not being able to use visual aids, and the inability to assess participants’ nonverbal cues. Providers used strategies for telephone encounters, such as high-level messaging to ensure the critical elements of the results were understood, and when possible, scheduling a follow-up in-person appointment to review more specific details. Some submitters incorporated teach-back in their telephone counseling. Lastly, for one case, the provider used telemedicine to accommodate the participant’s vision impairment.

### 3.3. Stakeholder Consultations

Six CSER consortium patient/community stakeholders, representing four CSER projects, provided feedback on the project goals and shared insights on the themes, challenges, and strategies identified in two cases selected from the case set. The six stakeholders were parents of children with genetic conditions (2), patients with a genetic diagnosis (1), patient/family advocates (2), and a community advocate (1). Five stakeholders were female, and one was male. The self-reported race/ethnicity of stakeholders were Black (4), White (1), and Latinx/Hispanic (1). Topics and recommendations from patient/community consultations are presented in Table 3.

Stakeholders highlighted the importance of addressing participants’ expectations up-front and helping patients/families identify questions they may have. They suggested using direct questions to assess expectations (for example, “What are you hoping to gain?”) and proposed providing patients with information about clinical genetics, the testing, and what to expect in the form of a frequently asked questions (FAQ) sheet before the return of results appointment. Stakeholders thought these strategies could reduce anxiety and help patients/families identify what they do and do not know to trigger relevant questions.

The stakeholder group stressed the potentially complicated nature of genomic results. Suggested strategies to make genomic information more manageable for participants included breaking up result disclosure into an initial and follow-up session, communicating information in multiple ways to accommodate visual and auditory learning styles, using lay language, incorporating teach-back to assess understanding, and providing reliable and appropriate educational resources. Stakeholders suggested that patients/families be encouraged to bring a trusted friend or advocate for the return of results appointment. This support individual, who is less emotionally attached to the results, may retain more of the information conveyed during the session and be more capable of identifying relevant questions.

Stakeholders recognized that disclosing results through a medical interpreter can impact how effectively information is conveyed and noted that specific genomic terms might not accurately translate to other languages. Compiling commonly used genomic terms and cross-checking the translations with a medical interpreter prior to the results disclosure was suggested to ensure appropriate and understandable language is used. In addition, stakeholders suggested consulting with the medical interpreter prior to the session regarding their comfort with and knowledge of genetics concepts.

Stakeholders emphasized that the patient/family-provider relationship is essential in helping clients adapt to their genomic results. They noted that establishing rapport during a relatively short interaction is difficult and suggested that conveying empathy and exploring what is specifically important for that particular patient could help. They also suggested maintaining the same care team for pre- and post-test counseling. Additionally, it was recommended that providers could be most effective when they work within a patient’s belief system(s). For example, within the context of religious faith, stakeholders stressed that a patient’s faith plays a vital role in their adaptation to their results and suggested involving a patient’s faith advisor to discuss results.

Finally, the psychological aspects of results disclosure were discussed. Stakeholders suggested that emotional responses, such as grief about the loss of the ‘perfect’ child or denial that a genetic condition is causing health problems, can complicate adaptation. The stakeholders’ general perspective was that it would be beneficial to involve more individuals than just the provider disclosing results to support the patient/family in their emotional needs. For example, a ‘support team’ might include the pediatrician/primary care physician, a community member, a faith advisor, and a genetic counselor. Finally, stakeholders highlighted the value of connecting the patient/family to other individuals or families in similar situations for additional support after the result visit.

## 4. Discussion

This set of representative results disclosure cases from the CSER consortium with accompanying themes, challenges, and strategies highlights recent experiences returning genomic results to participant populations that include diverse racial/ethnic and medically underserved patients in varying clinical research contexts. The themes identified in this case set emphasize areas of consideration for the practice of genomic medicine.

Low health literacy and language discordance were common themes perceived by providers to influence understanding in this diverse cohort. In contrast, providers in the first phase of CSER, when access to genomic sequencing was more limited, did not highlight these themes related to results disclosure [5]. Only 12% of adults in the United States have proficient health literacy, and low health literacy is more prevalent amongst medically underserved groups [14]. Universal health literacy precautions recommend assuming all patients may have trouble understanding health information and simplifying communication for all patients to reduce misunderstandings [15]. Applying these recommendations is essential to attend to patient needs. Training programs for health professionals and integrating health literacy into medical practitioners’ curricula can help to increase provider awareness and communication skills [16,17]. Stakeholders suggested developing FAQ sheets to educate patients/participants prior to results disclosure. The CSER NCGENES project is currently assessing whether this type of FAQ sheet improves patient outcomes, which may provide useful evidence for adopting this strategy more broadly.

As the practice of genomic medicine expands, providers may increasingly encounter language discordance with their patients and will need to communicate through medical interpreters. Providers may benefit from additional training on working with interpreters to ensure information is delivered equitably to all patients. Stakeholders suggested strategies to increase the quality of results disclosure sessions using interpreters, such as verifying that interpreters have adequate knowledge of genetic terminology prior to the visit. Resources that provide genomic training for interpreters are currently available [18], but it remains unclear whether such training is utilized or improves results disclosure sessions and patient understanding. The CSER Cancer Health Assessments Reaching Many (CHARM) project is exploring differences in medical interpretation during results disclosure based on exposure to a genetics training course, but additional research in this area is necessary.

The range of possible results and the abstract concepts involved when communicating the often complex, unexpected, or ambiguous results from genomic sequencing tests were perceived to impact participant understanding, even though providers often had several years of experience communicating such results. Strategies suggested by stakeholders to address the complexity of genomic results and aid participant understanding—multiple result conversations, sharing information in numerous ways, implementing teach-back—have been previously proposed [6]. Several CSER consortium projects have also incorporated these communication strategies into their study design, which may provide additional evidence on their effectiveness and support applying these strategies in clinical care. Incorporating a two-visit result disclosure model may provide additional support for patients who are overwhelmed by unexpected or complex results; however, it requires additional patient and provider time and resources. Novel approaches to support a two-visit model, such as utilizing telehealth solutions, may provide practical alternatives. Telehealth addresses access barriers related to travel for in-person visits, but it is essential to ensure patients without access to this technology can still receive appropriate care.

Setting realistic expectations for genomic testing continues to emerge as an important theme. Stakeholder comments regarding the need to elicit specific questions and goals from each participant emphasize the continued relevance of assessing and managing patient expectations and tailoring content to patient-specific needs. The importance of setting patient expectations is a key element of genetic counseling practice [19,20], and previous research on informed consent for genomic sequencing has stressed the importance of doing so during pre-test conversations [21]. Achieving this goal may require readdressing expectations throughout the testing process as expectations can change in the time between consent and results disclosure, which can span from weeks to many months.

Genomic results can provoke an emotional response, such as anxiety or disappointment, which may affect participants’ understanding, adaptation to the results, and ability to ask relevant questions in real-time. Stakeholders suggested that the presence of a “support team” during the visit could be beneficial. The value of using a team-based care model has been well described [22]. While clinical genetics practice often employs a team-based approach, rarely are members of the broader team present at the results disclosure session, precisely when stakeholders suggest a gap in support exists. Inviting patient-identified support individuals to results disclosure sessions may help address this gap.

Providers cited the highest number of cases with challenges related to the objectives “*Effectively educate clients about a wide range of genetics and genomics information based on their needs, their characteristics and the circumstances of the encounter” and “Identify, assess, and empathically respond to stated and emerging concerns*.” These challenges included limited dialogue during results disclosure, lack of concrete results information, and participants’ health literacy level. Strategies cited by providers to address these challenges reflect traditional genetic counseling practice (normalization, acknowledgment, affirmation of concerns, and providing information multiple ways), as well as modified results disclosure approaches that are incorporated into CSER consortium research protocols (education/communication tool, genetic counseling model that includes lay language, reducing the amount of information, and teach-back) [23,24]. Findings from CSER projects studying the implementation of these modified approaches will inform their utility to support achieving these objectives.

Recent work reported that a genetic counselor’s cultural competency level could positively or negatively impact rapport [25]. In this case set, providers did not report challenges related to the objective “*Apply genetic counseling skills in a culturally responsive and respectful manner to all clients.*” It is possible challenges were not cited because it is difficult, if not impossible, to self-assess one’s cultural competency. Exploring patient and/or third-party perspectives would likely provide more truthful insights into whether this objective is adequately met. Stakeholders also emphasized the importance of awareness of a patient’s cultural background and personal perspective to establish and maintain rapport. Given the importance of this objective, ongoing evaluation and training for providers working with culturally diverse patients/participants is critical, and diversifying the cultural makeup and linguistic capacity of genetic providers should continue to be an utmost priority [26].

There are several limitations to this study. The findings presented here represent a subset of providers’ perceptions and may not reflect the actual patient experience. We did not elicit the perceptions of CSER consortium participants, nor did we quantitatively assess participant health literacy and psychological outcomes for this project. Future research efforts should build on the provider impressions described here by exploring identified themes amongst patients/participants obtaining genomic testing. These findings are based on a limited case set and likely do not reflect a representative cross-section of all genomic results disclosure practices. Results disclosure conversations took place in the context of genomics studies with specific research questions; therefore, some of the themes, challenges, and strategies may be related to the study design and/or limitations of the research. Additionally, some themes and challenges were likely encountered more often due to the deliberate outreach and enrollment of underrepresented participant populations in CSER projects, while others commonly arise with all patients in the context of receiving a genomic test result. Additionally, the application of the genetic counseling PBCs to frame challenges and strategies in the context of genomic sequencing studies is outside of their intended use, which is to guide the training and assessment of genetic counselors [13]. Finally, patient/community stakeholders shared important feedback. However, only two cases were presented, and the stakeholder group included individuals with significant exposure to genomic medicine and research, which means their impressions may not be generalizable across a broader range of patient experiences.

## 5. Conclusions

The practice of genomic medicine will continue to evolve as access to genomic testing increases and challenges delivering complex genomic information persist. This project reports themes, challenges, and strategies that shape the delivery of genomic sequencing results in diverse racial/ethnic and medically underserved populations across a range of clinical contexts. Findings suggest that the quality of the patient-provider interaction and the complexity of genomic results are barriers to achieving results disclosure objectives; tailoring the discussion to individual patient needs may help address these challenges. Patient/community stakeholders provided a unique and valuable perspective on potential strategies for enhancing the patient experience, and their recommendations may help guide changes in practice. This paper adds to the knowledge base on results disclosure in the era of genomic medicine, and provides a valuable perspective on how to adapt and evolve disclosure practices to best serve diverse participant and patient populations.

## Figures and Tables

**Table 1:** Description of cases with related themes.

CSER Study Context				Germline Result(s)	Mode of Delivery	Themes
Project Name	Age	Phenotype	Germline Test			
SouthSeq	Newborns	Suspected genetic conditions	GS	VUS	In-person	Low health literacy; high-level genetics concept complicated understanding; disclosure by a non-genetic provider; parental distress
				VUS	In-person	Low health literacy; parental condition (maternal intellectual disability); ambiguous, discordant results complicated understanding and increased anxiety
				VUS	In-person	Low health literacy; ambiguous results complicated understanding; parental distress
P^3^EGS	Prenatal and Pediatric	Developmental delay ± congenital anomalies, structural anomalies in utero	ES	P variant (diagnostic)	Telephone/In-person	Language discordance between participant and provider; logistical challenges (distance, timing of return, due to pregnancy); competing medical priorities (indication for testing)
				1 LP and P variants (diagnostic)	Telephone/In-person	Low health literacy; language discordance between participant and provider; gauging parent understanding and coping
				VUS	In-person	Low health literacy (understanding instructions for sample collection); parental condition (maternal intellectual disability); logistical challenges (distance from the medical center, long work hours); ambiguous, complex results complicated understanding, Language discordance between participant and provider
KidsCanSeq	Pediatric	Cancer	ES	P variant (diagnostic)	Telephone	Language discordance between participant and provider; parental condition (vision impairment); mode of delivery (telephone ROR); gauging parent coping
NYCKidSeq	Pediatric	Suspected neurologic, immunologic, and cardiac genetic conditions	GS, neurodevelopmental panel	4 VUS	In-person	Low health literacy; language discordance between participant and provider (gauging parent understanding and coping)
			GS, neurodevelopmental panel	Chromosomal aneuploidy (incidental finding)	In-person	Overwhelmed by unexpected health issues; pre-test counseling (managing expectations); unmet expectations; family communication issues
			GS, neurodevelopmental panel	VUS	In-person	Low health literacy; language discordance between participant and provider; distrust in the medical system; complex results
			GS, immunodeficiency panel	Negative	In-person	Low health literacy; disappointed/unmet expectations; overwhelmed by the number of appointments
NCGENES2	Pediatric	Developmental disabilities, dysmorphology, neuromuscular disorders	ES	2 LP variants and 3 VUSs	Telephone/In-person	Low health literacy; multiple, complex results complicated understanding; family testing coordination
				2 VUSs	In-person	Loss of hope; peace with support organization; parental distress
				2 VUSs	In-person	Complex, unexpected results; incorporating new information; managing expectations
				Homozygosity for 3 VUSs	In-person	Complex, unexpected results; ambiguous results led to unanswered questions; parental distress; access to sequencing and medical/family history (adopted child)
CHARM	Adult	Hereditary cancer risk	ES	P variant (incidental finding)	Telephone	Low health literacy; low numeracy complicated understanding
				Normal	Telephone	Language discordance between participant and provider; participant anxiety complicated incorporation of new information; importance of contracting and checking comprehension

Abbreviations: GS, genome sequencing; ES, exome sequencing; P, pathogenic; LP, likely pathogenic; VUS, variant of uncertain significance.

**Table 2:** Case challenges and strategies for each result disclosure objective.

Objective	1. Establish a Mutually Agreed Upon Genetic Counseling Agenda	2. Identify, Assess, and Empathically Respond to Participant Concerns	3. Facilitate Informed Decision Making and Adaptation To Genetic Risks	4. Effectively Educate Clients About a Range of Genetics/Genomics Information	5. Apply Genetic Counseling Skills in a Culturally Responsive and Respectful Manner	6. Adapt Genetic Counseling Skills for Varied Service Delivery Models
Total Cases Citing Objective	6	11	7	13	2	8
Related Challenge (N cases)	Unmet expectations Unanswered questions Participant not ready to receive results, due to conflicting health concerns (3 cases)	Participants distressed and/or feeling overwhelmed Language discordance between participant and GC and/or lack of dialogue made it difficult to assess emotional needs Lack of trust in the medical system Participant not ready to receive results, due to conflicting health concerns Assessing whether anxiety adequately addressed (6 cases)	Lack of understanding of complex concepts Lack of concrete information with uncertain results Unexpected findings Ambiguous findings Confusion about clinical vs. research results Uncertainty about the inheritance of the variant, due to missing parental sample (7 cases)	Complicated by an emotional situation Lack of concrete information with uncertain results Low health literacy Language discordance between participant and GC (8 cases)	(0 cases)	Conveying complex concepts by phone Cannot use visual aids Cannot evaluate nonverbal cues (3 cases)
Potential Strategy (N cases)	Contracting Elicit expectations, perceptions, knowledge, and concerns Ask direct questions (3 cases)	Acknowledge, affirm and address concerns Active listening Retain psychosocial aspects between appointments Evaluate individual experiences and beliefs Focus discussion on addressing concerns Normalize to mitigate guilt (7 cases)	(0 cases)	Provide information in different ways, multiple times Provide personalized booklet Use an educational tool Literacy-focused genetic counseling approach Follow-up calls to reassess understanding (5 cases)	Consistency amongst the research team Establish rapport Use of an interpreter Prioritize the needs/wants of the parent (2 cases)	Telemedicine offered to meet the family’s needs and accommodate a disability High level messaging via telephone Follow-up with an in-person appointment if possible Incorporated teach-back to assess understanding via telephone (6 cases)

**Table 3:** Topics and recommendations from patient/community stakeholder consultations.

Topic	Stakeholder Recommendations
Complex Genetic Information	Consider multiple disclosure visits Use several communication approaches Incorporate teach-backUse lay language Provide reliable educational resources
Patient Expectations	Share a pre-visit FAQ sheet Ask direct questions
Patient/Family—GC/Provider Rapport	Provide a consistent care team pre and post-test Convey empathy Explore patient-specific goals Incorporate patient’s faith (if applicable) Build awareness of patient’s cultural background
Working with Medical Interpreters	Verify translations pre-visit Compile and share translations of genomics terms Consult pre-visit about comfort with and knowledge of genetic terms
Emotional Response/Adaptation	Include members of a support team Connect to patients/families in a similar situation

## Data Availability

No new data were created or analyzed in this study. Data sharing is not applicable to this article.
